# The Inflamed Intestinal Barrier as a Formulation Variable in Oral Nanocarrier Design: Evidence-Informed Principles for Mucus Interaction, Epithelial Access, and Translational Testing

**DOI:** 10.3390/pharmaceutics18091132

**Published:** 2026-09-09

**Authors:** Zofia Śledzikowska, Karolina Żylińska, Natalia Makaruk, Dominika Kubicka, Filip Adam Błotniak, Napoleon Waszkiewicz

**Affiliations:** Department of Psychiatry, Medical University of Bialystok, Michała Wołodyjowskiego 2, 15-272 Białystok, Poland; 37519@student.umb.edu.pl (K.Ż.); 41449@student.umb.edu.pl (N.M.); 38866@student.umb.edu.pl (D.K.); 41285@student.umb.edu.pl (F.A.B.); napoleon.waszkiewicz@umb.edu.pl (N.W.)

**Keywords:** oral nanocarriers, inflammatory bowel disease, intestinal mucus, epithelial barrier, diseased-tissue selectivity, biomolecular corona, translational benchmarking, nanomedicine

## Abstract

Oral nanocarrier development for inflammatory bowel disease (IBD) is complicated by inflammation-dependent changes in mucus, epithelial integrity, and immune-cell interactions. This critical narrative review examines comparative evidence from in vitro systems, animal models, and human intestinal tissue, alongside clinical studies, to assess how barrier state modifies formulation performance. Across the reviewed studies, particle size alone does not consistently predict lesion accumulation, while surface chemistry, coating, geometry, and stability in gastrointestinal fluids influence mucus interaction, cellular access, and payload release. Tissue-associated fluorescence, increased permeability, and cellular uptake do not independently establish intact-carrier transport or productive delivery. Clinical trials of selected oral nano-enabled formulations report improvements in some disease outcomes but do not establish inflammation-selective delivery as the underlying mechanism. We propose an evidence-informed benchmarking framework linking characterization in biological media to mucus transport or retention, epithelial–immune responses, spatial localization, target engagement, and safety. Matched healthy and inflamed comparators are central to testing selectivity, and preclinical findings must be distinguished from clinical validation. Artificial intelligence may support barrier-state-aware formulation optimization but requires standardized datasets and prospective experimental validation. Given the heterogeneous evidence base and limited direct human delivery studies, prospective validation should prioritize local payload delivery and therapeutic benefit without exacerbating intestinal barrier injury.

## 1. Introduction

Oral nanocarriers are being developed to protect labile payloads, alter regional exposure, prolong mucosal residence, and reduce systemic toxicity in inflammatory bowel disease (IBD). Investigated platforms include polymeric and lipid nanoparticles, nanocrystals, nanoemulsions, biomimetic carriers, and plant-derived nanovesicles [1,2,3,4,5,6,7,8,9,10,11,12,13,14]. Recent reviews emphasize complementary approaches: exploiting disease-associated changes in the gastrointestinal environment, using reactive oxygen species (ROS)-responsive materials, and incorporating biological membranes or vesicular components to influence carrier–host interactions [3,4,5]. These approaches connect formulation design with the local inflammatory microenvironment rather than with anatomical targeting alone. However, regional release, inflammation-responsive activation, and cell-specific delivery represent distinct claims. In this review, precision nanotherapeutics are therefore evaluated according to whether the proposed targeting mechanism is supported by differential exposure, activation, or functional delivery under defined biological conditions. Physicochemical descriptors remain necessary for product definition but cannot independently establish therapeutic selectivity.

The intestinal barrier is a sequence of coupled environments rather than a single obstacle. A carrier encounters saliva, gastric and intestinal fluids, dietary components, enzymes, and microbial products before contacting mucus; it may then be retained, diffuse, aggregate, release its payload, associate with epithelial cells, undergo immune sampling, or cross damaged tissue [12,15,16,17,18,19,20,21,22,23,24]. Each transition can change particle identity. Hydrodynamic diameter and zeta potential measured in water describe the starting formulation, not necessarily the object presented to inflamed tissue.

Inflammation changes the delivery problem. Mucin abundance, glycosylation, hydration, continuity, and viscoelasticity may differ by disease, segment, lesion, and treatment. Epithelial erosion and altered tight-junction organization change paracellular flux, while immune-cell recruitment and inflammatory proteins change binding, uptake, and tolerability [15,16,17,18,19,25]. Consequently, stronger tissue-associated signal can represent useful lesion retention, nonspecific trapping in ulcer debris, uptake by phagocytes, passage through denuded epithelium, or systemic leakage. These mechanisms have different therapeutic and safety implications.

A recurring weakness in the literature is the conversion of context-dependent observations into universal design rules. Small particles are often described as mucus-penetrating, cationic particles as mucoadhesive, dense PEG as stealthy, and inflamed tissue as passively permeable. Available comparative studies challenge these generalizations but do not establish a replacement set of universal rules. Different size trends have been reported in animal and human studies conducted using different routes and materials [26,27]; surfactant or ligand chemistry has altered performance in specific preclinical systems [28,29,30]; and a comparison in human intestinal tissue identified preferential uptake of wormlike micelles relative to composition-related spheres and vesicles [31].

This review prioritizes comparative and functional evidence to link formulation variables with delivery outcomes. It distinguishes direct intestinal findings from extrapolations based on synthetic gels or non-intestinal mucosa, and quality-control conventions from IBD-specific predictors. Section 6 translates this synthesis into proposed research criteria requiring prospective validation.

## 2. Literature Identification and Critical Synthesis

This article is a focused critical narrative review, not a systematic review or meta-analysis. The initial targeted literature identification covered publications available through 27 July 2026, using PubMed as the primary bibliographic database. Additional literature was incorporated during manuscript revision, including reviewer-suggested sources; these additions should not be interpreted as an exhaustive database update. Searches combined concept blocks for intestinal inflammation or IBD with oral nanoparticles or nanocarriers and one or more of the following: mucus, mucoadhesion, penetration, particle size, geometry, polydispersity, zeta potential, PEG, hyaluronic acid, protein corona, epithelial permeability, organoid, co-culture, ex vivo tissue, clinical trial, and safety. Additional records were identified from reference lists of relevant primary studies and recent reviews. The search was iterative because the objective was mechanistic integration rather than exhaustive frequency estimation. The concept blocks above summarize the search scope, not a single reproducible Boolean strategy. Search-by-search strings, screening counts, and independent duplicate selection are not documented here; consequently, the completeness and reproducibility of literature identification remain limited.

Primary studies were prioritized when they met at least one of four criteria: (i) direct comparison of healthy and inflamed intestinal conditions; (ii) comparison of formulations differing in a defined physicochemical property; (iii) spatial or cell-specific localization beyond bulk fluorescence; or (iv) a functional endpoint such as payload release, target engagement, barrier recovery, cytokine modulation, or safety. These were narrative prioritization criteria, not a prospectively registered eligibility algorithm. Oral clinical trials were considered for product-specific efficacy and tolerability; rectal human studies and ex vivo human tissues were considered separately for localization and mechanism. Animal and in vitro studies informed mechanistic hypotheses and proposed tests, not clinical advancement criteria. Evidence from non-intestinal mucus, other diseases, or non-oral routes was treated as indirect for oral IBD delivery rather than excluded from mechanistic discussion. Reviews were used to map the field and identify studies, but quantitative formulation claims were anchored to primary evidence whenever possible.

Evidence was interpreted across five linked domains: identity in biological media, mucus interaction, epithelial access, immune response, and translational confirmation. We explicitly distinguished particle association from uptake, uptake from translocation, translocation from productive payload delivery, and local exposure from therapeutic selectivity. Because study designs, disease models, doses, materials, and readouts were heterogeneous, no pooled effect estimate was attempted. No PRISMA flow diagram or formal risk-of-bias score is presented; this limitation is retained to avoid implying systematic completeness. For the core comparisons, Table 1 records the study, model/comparator, formulation variable, observation, and defensible interpretation; Table 2 links formulation recommendations to representative sources and their experimental settings. Interpretation considers whether the route, barrier state, comparator, and measured endpoint directly address the claim. Neither a positive disease outcome alone nor a human-derived model upgrades the evidence for an unmeasured delivery mechanism. The synthesis distinguishes observations within the tested system from mechanistic extrapolations and author-proposed benchmarking criteria. Direct human delivery evidence is limited: rectal particle-localization studies [27] and ex vivo intestinal-tissue comparisons [31] address specific mechanisms, while patient-derived organoids [32] remain experimental models rather than clinical delivery studies. The oral clinical trials assess selected products but do not validate the proposed barrier-state-aware sequence [33,34,35]. Consequently, the framework is hypothesis-generating; neither the narrative synthesis nor the proposed criteria constitute a formal evidence grade or validated predictor of clinical success.

Evidence provenance is described using distinct categories: human clinical trials (patient outcomes after treatment); human in vivo mechanistic studies (localization or exposure in participants); ex vivo human tissue studies; animal in vivo studies; ex vivo animal tissue or mucus assays; in vitro cellular studies, including patient-derived organoids; and acellular or computational mechanistic studies. Reviews and analytical or regulatory guidance are identified separately from primary experimental evidence. These categories describe the source and directness of support, not a formal certainty hierarchy: an ex vivo human study may directly address tissue localization without establishing clinical efficacy, whereas a clinical trial may demonstrate a product-specific outcome without establishing barrier-state selectivity. No formal risk-of-bias assessment or certainty grading was performed, and selection and publication bias cannot be excluded. The reporting and advancement recommendations remain author-proposed syntheses, even when several evidence categories support their rationale.

## 3. Inflammation Changes the Formulation Problem

### 3.1. Luminal Preconditioning and Mucus Remodeling

Before reaching the colon, an orally administered nanocarrier is exposed to saliva and gastrointestinal fluids that can change its colloidal and interfacial properties. Salivary proteins, mucins, electrolytes, enzymes, immunoglobulins, and microbial products may promote aggregation or corona formation and may mask targeting ligands [21,22,23,24]. We propose a characterization sequence, adapted to the formulation and intended release site, that assesses size distribution, PDI, surface potential, ligand accessibility, and payload retention after sequential exposure to saliva-relevant, gastric, intestinal, and mucus-containing media. These tests assess transformation during transit but do not reproduce the full oral route. For enteric-protected systems, this sequence applies first to the complete dosage form and then to the nanoparticles liberated at the intended release site (Section 4.4).

Mucus is simultaneously a clearance system, a diffusion barrier, and a potential depot, but its properties differ between active disease, remission, and health. Changes in IBD involve not only mucus abundance but also mucin production and post-translational processing. The systematic review by Arras et al. identified reduced MUC2 protein despite unchanged mRNA expression, together with abnormalities in glycosylation and sulfation [60]. These compositional changes may alter mucin–particle interactions, but their net effect on carrier mobility requires direct measurement rather than prediction from mucin expression alone. Active inflammation may also produce spatially heterogeneous mucus disruption, with preserved regions adjoining erosions or ulcers [15,16,17].

Importantly, clinical or endoscopic improvement does not invariably restore mucus barrier function. In human sigmoid-colon biopsies, Johansson et al. demonstrated increased mucus penetrability in active ulcerative colitis (UC) and in a subgroup of patients in endoscopic remission [61]. Thus, quiescent UC should not automatically serve as a healthy-mucus comparator. For nanocarrier studies, this observation supports measuring native mucus composition, penetrability, and particle mobility alongside local disease activity. Retention and penetration should be evaluated separately in active and quiescent disease.

The classic rat study by Lamprecht et al. illustrates inflammation-dependent deposition. After oral administration, 0.1-µm particles showed 14.5 ± 6.3% deposition in inflamed colon compared with 2.2 ± 1.6% in controls; 1-µm particles showed 9.1 ± 4.2% versus 2.0 ± 0.8%, and 10-µm particles 5.2 ± 3.8% versus 1.4 ± 0.6% [26]. The gradient supports preferential retention of small particles in this model, but it does not establish a universal optimal diameter or identify whether fluorescence resided in mucus, ulcer material, epithelium, or immune cells.

Human evidence complicates a simple “smaller is better” rule. In 33 patients with IBD and six controls, Schmidt et al. rectally administered 250-nm nanoparticles and 3.0-µm microparticles. Microparticles accumulated in ulcerated lesions (median covered area reported as 1.28%, range 0.83–3.45) but were absent from control mucosa; 250-nm particles were detected only as traces [27]. Ex vivo experiments suggested persorption at discontinuities rather than uniform diffusion. Differences in route, particle composition, sampling, and endpoint limit direct comparison with the rat study.

### 3.2. Epithelial Disruption and the Meaning of Apparent Transport

The epithelial barrier restricts macromolecular flux through cell membranes, tight junctions, mucus, and immune-sampling routes. Inflammation can reduce TEER, redistribute claudins, occludin, and ZO-1, increase cell shedding, and create focal erosions [15,19,62]. None of these changes creates a uniform pore-size sieve. Apparent passage of 100–200-nm particles across an inflamed monolayer should not be described as paracellular diffusion through normal tight-junction pores. More plausible alternatives include transcellular uptake, passage at damaged regions, M-cell or immune sampling, or experimental leakage [19,63,64].

During remission, epithelial restitution and junctional recovery may reduce permeability, but symptom control, endoscopic healing, histologic remission, and functional barrier restoration are not interchangeable endpoints. In the prospective ERIca study, 181 patients with clinically remittent IBD underwent assessment of barrier function by confocal endomicroscopy. Barrier healing predicted freedom from major adverse outcomes more effectively than endoscopic or histologic remission [65]. This clinical evidence does not establish nanoparticle permeability, but it demonstrates why apparently quiescent tissue cannot be assumed to have normal barrier function. Consequently, comparisons of carrier transport should document local epithelial integrity and distinguish clinically inactive disease with residual barrier defects from objectively healed mucosa and non-IBD controls. Otherwise, differences attributed to formulation design may instead reflect incomplete tissue recovery.

TEER is particularly vulnerable to overinterpretation. A decrease can confirm barrier disruption but does not localize the carrier or prove that an intact nanoparticle crossed. Fluorescent labels may dissociate, payload may diffuse independently, and transwell recovery may be incomplete. Mechanistic transport studies should combine TEER with size-appropriate paracellular tracers, tight-junction imaging, mass balance, label-stability controls, and orthogonal quantification of the carrier and payload. Spatial microscopy should distinguish apical mucus, epithelial membrane association, intracellular signal, basolateral appearance, and uptake by immune cells.

A human ‘leaky gut’ model demonstrates why uptake and target engagement must be separated. Hartwig et al. used a Caco-2/THP-1/MUTZ-3 system with regions below 200 Ω·cm^2^ and observed accumulation of si*JAK1* nanoparticles in subconfluent areas and immune cells. Nevertheless, functional target engagement was limited (approximately 10 ± 12% to 16 ± 12%) compared with 86 ± 2% for tofacitinib [37]. The model therefore detected access without assuming effective intracellular delivery.

### 3.3. Immune Environment, Corona Formation, and Tolerability

Active intestinal inflammation changes both the abundance and the functional state of mucosal immune cells. Neutrophil recruitment, inflammatory monocyte/macrophage accumulation, and altered lymphocyte populations create a cytokine-, enzyme-, and reactive oxygen species (ROS)-rich environment [15,18,66]. As a human example, single-cell profiling by Smillie et al. identified expansion of inflammatory monocytes and CD8+ T cells expressing IL-17, together with inflammatory fibroblasts, in UC mucosa [67]. These findings indicate that the cellular environment encountered by a carrier is heterogeneous rather than a uniform population of activated macrophages. Increased phagocyte uptake may represent productive delivery, sequestration, or degradation; these outcomes require separate functional readouts.

With histologic resolution, acute neutrophilic activity subsides, but clinical remission alone does not establish normalization of immune-cell composition or local inflammatory stimuli [15]. Formulations relying on ROS, inflammatory enzymes, or phagocyte uptake therefore require evaluation under both active and quiescent conditions, particularly if intended for maintenance treatment. Inflammatory proteins, extracellular DNA, and damaged-cell products may additionally modify carrier adsorption, opsonization, and degradation [4,5,18,23,24,66,68]. The consequences of these interactions for biomolecular corona formation and biological identity are discussed in Section 3.6.

Myeloperoxidase (MPO) is a positively charged inflammatory protein and is frequently invoked as a mechanistic rationale for anionic carrier retention [68]. Charge complementarity alone, however, does not demonstrate MPO-mediated targeting. A direct claim requires competitive binding, MPO depletion or inhibition, colocalization, or another perturbation that separates MPO binding from nonspecific interaction with cationic lesion components. Until such experiments are performed, MPO should be presented as a plausible contributor to electrostatic retention, not as an established universal targeting mechanism.

Inflammation also changes safety. In a three-cell intestinal co-culture, inflammatory stimulation increased sensitivity to some nanomaterials and altered IL-8 responses [38]. Other inflamed co-culture work similarly indicates that baseline inflammation can amplify toxicity [25,69]. This means that a carrier appearing inert in healthy epithelial monoculture may still aggravate cytokine release, oxidative stress, or barrier loss under disease conditions. Silica nanoparticles have exacerbated intestinal inflammation after oral exposure in vivo [70], underscoring the need to measure barrier recovery and immune response, not merely cell viability.

### 3.4. Extracellular Matrix Remodeling and Persistent Fibrosis

Extracellular matrix (ECM) remodeling adds a tissue-level dimension that is not captured by mucus or epithelial permeability alone. Repeated injury and repair can promote collagen-rich matrix accumulation, fibroblast activation, and increased tissue stiffness, particularly in fibrostenotic Crohn’s disease (CD). Johnson et al. measured increased stiffness in human Crohn’s strictures and showed that matrices reproducing pathological stiffness induced a fibrogenic phenotype in cultured human colonic fibroblasts [71]. More recently, Zhang et al. identified enrichment of ECM-producing FAP+ fibroblasts in fibrotic human ileal CD tissue and implicated TWIST1-dependent signaling in complementary mechanistic experiments [72].

Fibrosis should therefore be treated as a partly distinct axis from current inflammatory activity: suppression of a flare does not establish reversal of accumulated matrix or restoration of normal tissue mechanics. For oral delivery, the subepithelial ECM becomes relevant when the carrier or released payload must reach stromal or deeper immune targets after traversing an intact or disrupted epithelial surface. Dense or remodeled matrix could influence distribution and retention, but these fibrosis studies do not directly demonstrate nanocarrier transport. Accordingly, antifibrotic delivery claims require localization within fibrotic tissue and functional target engagement, rather than extrapolation from epithelial uptake in acute colitis.

### 3.5. Microbiota Alterations and Regional Heterogeneity

Disease-associated changes in microbiota composition are accompanied by changes in microbial function and metabolites. In a longitudinal multi-omics study, Lloyd-Price et al. observed increased facultative anaerobes, depletion of obligate anaerobes, and altered bile-acid and short-chain-fatty-acid profiles during dysbiotic periods in IBD. Reduced butyrate was accompanied by depletion of organisms such as *Faecalibacterium prausnitzii* and *Roseburia hominis*, while periods of disease activity showed greater temporal instability [73]. These findings argue against assigning all active or quiescent patients a single microbial profile. For microbiota-responsive carriers, a relevant implication is that cleavage or release should be measured in patient-derived or appropriately reconstructed microbial environments. Clinical remission alone should not be used as evidence that microbial enzyme activity has returned to a healthy reference state, and taxonomic composition cannot substitute for a direct release assay.

Regional anatomy further modifies these disease-state effects. Comparative experiments in mice demonstrated firmly attached mucus in the stomach and colon, contrasting with more readily removable, penetrable mucus in the small intestine [74]. These findings establish regional differences experimentally, although their quantitative properties should not be transferred directly to human IBD. Along the oral route, gastric acidity, small-intestinal digestive fluids, and the colonic microbial environment impose different preconditioning and release requirements [12,16]. Disease distribution adds another distinction: UC primarily affects the colonic mucosa, whereas CD may involve discontinuous gastrointestinal segments and extend through the bowel wall [15]. An actively ulcerated colonic site, a quiescent previously affected segment, and a fibrotic ileal stricture therefore represent different delivery environments.

For example, a carrier retained at exposed ulcer surfaces during active colitis may lose that retention mechanism after epithelial healing; this is a formulation hypothesis requiring longitudinal testing, not proof of reduced efficacy in remission. Likewise, successful colonic release does not establish delivery to an ileal CD lesion. Comparative experiments should therefore record the sampled gastrointestinal segment, local inflammatory and histologic activity, fibrosis, and treatment exposure, and distinguish non-IBD controls from noninflamed regions within an affected patient.

### 3.6. Biomolecular Coronas and the Evolving Biological Identity of Nanocarriers

The biological identity of an oral nanocarrier comprises its transformed structure, adsorbed biomolecules, accessible surface groups, and associated payload at a particular gastrointestinal location and time. Protein adsorption is dynamic: more persistent associations coexist with rapidly exchanging components, commonly described as hard and soft coronas, respectively [23,24]. Digestive enzymes, dietary proteins, mucins, and inflammatory proteins create exposure conditions that cannot be represented by serum incubation alone. Exposure history also matters. Brouwer et al. found that a digestion-associated corona on polystyrene nano- and microplastics persisted during subsequent incubation in serum-containing medium and altered macrophage uptake in a size- and charge-dependent manner [75]. Although this was an in vitro toxicology model rather than an IBD drug-delivery study, it demonstrates that sequential exposure may produce a different biological interface from direct contact with the final medium.

Corona formation is not necessarily detrimental. In male rats with colitis, Wu et al. linked nanoparticle surface hydrophobicity and rigidity to the composition of a colitis-specific intestinal protein corona. Greater hydrophobicity increased protein adsorption, whereas high rigidity favored enrichment of macrophage-associated targeting proteins, notably S100A8; the resulting budesonide-loaded formulations improved macrophage delivery and therapeutic outcomes [76]. This provides formulation-specific evidence that an acquired corona can contribute to targeting rather than merely concealing a synthetic ligand. It does not establish that hydrophobic or rigid particles are universally preferable, or that the same corona will form in human IBD.

A mucus corona should likewise be distinguished from simple entrapment within the mucus gel. The term refers here to mucins and other mucus-derived biomolecules associated with the particle surface, whereas bulk retention may occur without demonstrating such an adsorbed layer. Mucin association can change effective surface charge, promote particle clustering, and modify subsequent cellular trafficking. Yang et al. reported that intestinal mucin increased endocytosis of PEG-modified gold nanoparticles but reduced transcytosis across enterocytes, with nanoclustering and altered retrograde trafficking implicated [77]. This model illustrates why increased cellular uptake after mucus exposure cannot be equated with improved transepithelial delivery. Purified-mucin experiments provide mechanistic information but do not reproduce the full composition or inflammatory heterogeneity of native intestinal mucus.

Surface adsorption also occurs alongside structural transformation. Gastric acidity and changing ionic strength may alter protonation and aggregation, while intestinal enzymes and bile components can promote degradation, lipid digestion, or colloidal reorganization, depending on the material [12,21,22,23,24]. Lipid digestion may redistribute a hydrophobic payload into mixed micelles or other digestion-derived structures, so the absorbed species need not be the original nanocarrier. Zhan et al. developed a polarity-sensitive near-infrared-II probe to monitor lipid-nanocarrier lipolysis while reducing interference from free probe and mixed micelles [78]. This experimental approach highlights the need to distinguish carrier persistence from the persistence of a fluorescent label; its performance remains dependent on the formulation and biological environment in which it is validated.

Microbiota interactions are bidirectional: microbial activity may transform susceptible carrier components, while carriers and their cargo may influence bacterial growth, metabolism, and host signaling. Teng et al. demonstrated lipid-dependent uptake of ginger-derived exosome-like nanoparticles by Lactobacillaceae and linked their RNA cargo to altered bacterial metabolism and IL-22-dependent protection in mouse colitis [79]. This represents a microbiota-mediated mechanism rather than proof of direct epithelial drug delivery. Accordingly, bacterial association, microbial transformation, and changes in metabolites should be distinguished from host-cell uptake. Empty-carrier, free-payload, and microbiota-dependent controls are particularly informative when an anti-inflammatory effect could arise through either route.

Operationally, biological identity should be characterized after sequential gastrointestinal exposure and linked to function. Alongside size, aggregation, and payload retention, relevant measurements include carrier integrity, accessible ligands, and the composition of adsorbed biomolecules. Particle-free medium controls and validated separation procedures are needed to distinguish particle-associated material from free proteins, mucin aggregates, and endogenous colloids. Because washing can remove weakly associated components, the recovered corona should be described according to the isolation method. The decisive comparison is whether the transformed formulation changes mucus mobility, target-cell delivery, payload activity, or toxicity under matched healthy, active, and quiescent conditions—not simply whether additional proteins are detected.

## 4. Evidence-Informed Interpretation of Formulation Variables

### 4.1. Particle Size: A Task-Specific Variable, Not a Universal Threshold

**Figure 1:**
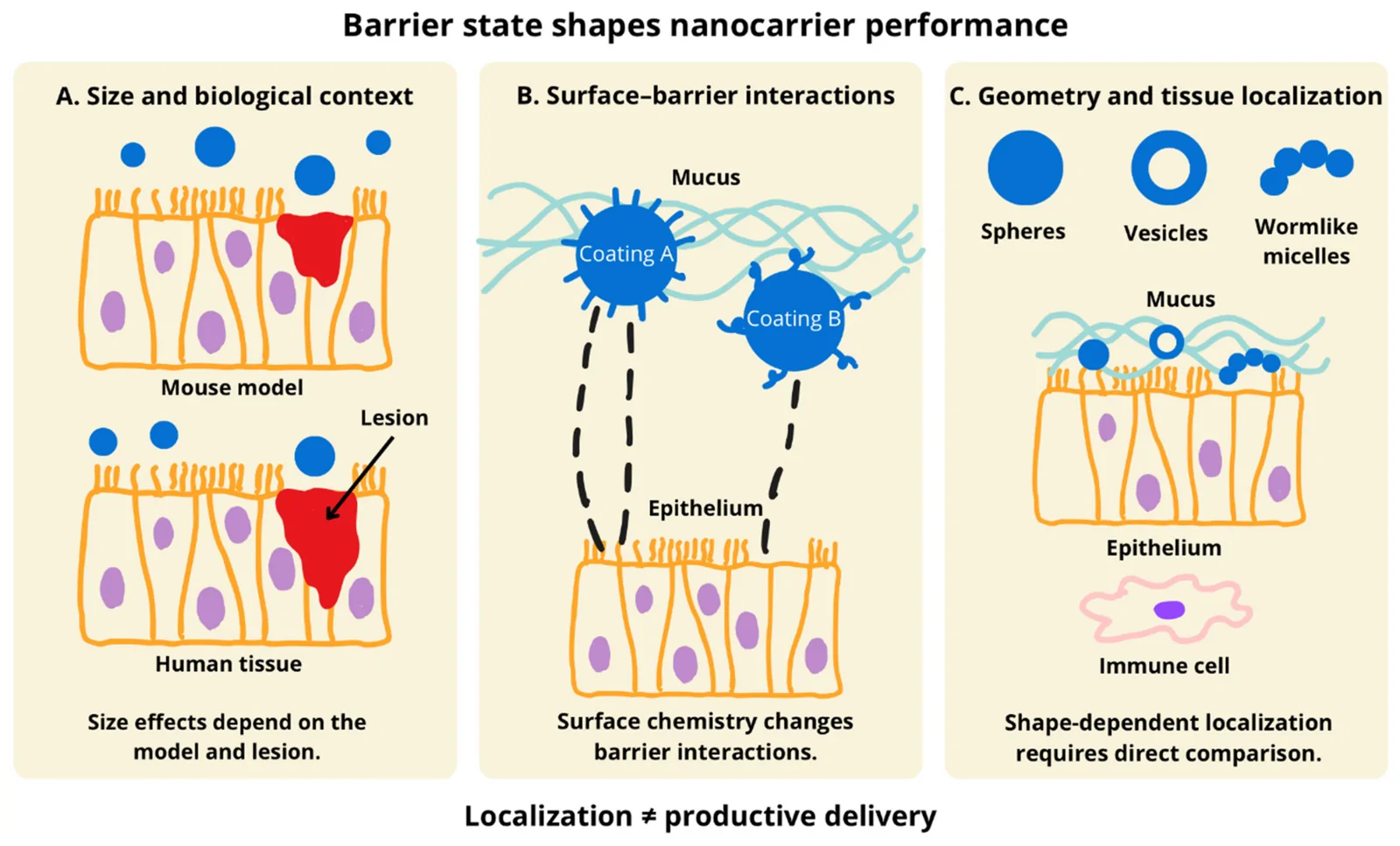
Barrier state shapes nanocarrier performance. Conceptual overview of context-dependent nanocarrier–barrier interactions. (**A**) Differently sized particles and schematic lesions in mouse models and human tissue illustrate the need to interpret size effects within the experimental setting. (**B**) Distinct surface coatings illustrate how interfacial chemistry can influence mucus interactions and epithelial access. Dashed paths are illustrative and do not denote validated transport routes or comparative transport rates. (**C**) Spheres, vesicles, and wormlike micelles illustrate geometry as a variable requiring direct comparison; mucus, epithelium, and an immune cell indicate relevant sites for localization assessment, without implying equivalent access by all shapes. The panels summarize concepts discussed in Section 4, not quantitative study results or universal rankings. Carrier localization alone does not establish productive payload delivery or therapeutic efficacy. Particle sizes, numbers, positions, and lesion dimensions are schematic and not to scale. “Coating A” and “Coating B” schematically represent alternative surface chemistries and do not denote specific coating materials.

Particle size influences diffusion, curvature, sedimentation, cellular uptake, drug loading, release, and manufacturability, but its effect is conditional on surface chemistry and the biological medium [12,18,20,40,49]. In mucus, diameter must be interpreted relative to the local mesh and to adhesive interactions. A nominally small cationic particle can be immobilized by mucins, whereas a larger, densely hydrophilic particle can move more freely. Intestinal mucus studies show that surface chemistry, lipid exposure, and sample structure alter apparent size selectivity [40,41,42]. Figure 1 summarizes size-, surface-, and geometry-dependent interactions.

The commonly repeated categories of <100 nm, 100–200 nm, and >200 nm can be useful for structuring experiments, but they are not validated clinical cutoffs for IBD. The rat–human comparison in Section 3.1 illustrates the limitations of cross-study size rankings [26,27]. For a mucus-penetrating task, the key endpoint is diffusion or distribution in native disease-relevant mucus. For lesion retention, spatial residence and washout matter. For a claim of intact-carrier epithelial access, localization is relevant; target engagement or payload activity is a separate endpoint when functional delivery is claimed.

Size distributions should be reported before and after biological exposure. Mean hydrodynamic diameter alone can conceal aggregates or a small subpopulation responsible for tissue penetration. Dynamic light scattering is intensity weighted and should be complemented by number-sensitive or imaging methods when heterogeneity is mechanistically relevant [49,50,51]. Electron microscopy documents morphology in a dry state; it does not replace hydrodynamic characterization. A credible report should state the analysis model, distribution metric, replicate number, medium, temperature, and stability interval.

### 4.2. Surface Charge and Coating: Measure Biological Identity

Cationic surfaces can interact with negatively charged sialylated and sulfated mucins and may increase retention. The same interaction can immobilize particles, increase nonspecific epithelial contact, and amplify toxicity. Anionic or near-neutral coatings may reduce mucoadhesion, but inflammation-associated proteins and receptors can create new retention mechanisms. The 34.8-fold inflamed-to-healthy difference reported for polysorbate-20-coated particles of approximately 200 nm supports the importance of interfacial chemistry within the tested murine system but does not establish its dominance over size across formulations or in human IBD [29].

Hyaluronic acid (HA) illustrates the need to distinguish chemistry from generic charge. HA-coated particles can interact with CD44 and other inflammation-associated pathways, yet their behavior depends on molecular weight, substitution, coverage, stability, and the underlying core. In inflamed Caco-2 cells, HA nanoparticles showed increased uptake consistent with higher CD44 expression and improved anti-inflammatory effects of budesonide [30]. In a colitis model, inflammation promoted retention of HA-functionalized mesoporous silica relative to PEG-functionalized particles, while biodistribution revealed organ exposure that would have been missed by a colon-only analysis [28].

PEG reduces hydrophobic and electrostatic adhesion when it forms a sufficiently dense, hydrated layer. Mushroom-like and brush-like conformations are determined by polymer molecular weight, grafting density, surface curvature, and competing ligands [44,45,80]. Dense PEG can improve mucus diffusion but can also reduce epithelial uptake and obscure targeting ligands. Reports should specify PEG molecular weight, surface density or a justified proxy, conjugation chemistry, ligand accessibility, and coating stability after gastrointestinal exposure. Evidence from airway, cervicovaginal, or synthetic mucus can support a transport mechanism [44,46,81] but should be labelled as indirect until confirmed in intestinal and inflamed mucus.

### 4.3. Geometry and Polydispersity: Plausible Mechanisms, Limited IBD Thresholds

Rod-like and wormlike particles can align with pores, undergo rotational diffusion, present greater contact area, and deform or entangle differently from spheres. Studies in mucosal tissues and polymer gels provide mechanistic support for geometry-dependent transport [47,48,82]. However, many such studies use non-intestinal mucus, synthetic gels, or cancer models. Their findings generate hypotheses for IBD rather than direct formulation rules. The human tissue study by Gardey et al. is therefore especially important: wormlike micelles selectively accumulated in inflamed tissue and immune cells, whereas composition-related spheres and vesicles did not show the same pattern [31]. This ex vivo observation supports further comparison of geometry while controlling composition, size, and flexibility where feasible; it does not establish a clinically preferred shape or prove that geometry alone explains the difference. Recent work also identifies deformability as a relevant variable. Lv et al. developed an epigallocatechin gallate delivery system based on a flexible Pickering emulsion stabilized by chitosan nanoparticles. Compared with rigid chitosan microspheres, the system showed improved mucus permeation and better outcomes in dextran sulfate sodium-induced acute colitis in mice [83]. This extends consideration beyond nominal size and shape, but the comparison involves different carrier architectures and does not establish flexibility as an isolated determinant of performance. Its relevance to human inflamed mucus remains to be tested.

Polydispersity index (PDI) is a quality and interpretability metric, not an IBD-specific biological threshold. Values below approximately 0.2 are often used as a practical indicator of a narrow distribution for lipid and polymer systems, but no PDI cutoff has been validated to predict inflamed-mucus transport, lesion selectivity, or clinical performance [49,50]. A high PDI can mask subpopulations with different clearance or uptake, while a low PDI does not ensure stability after exposure to proteins or mucins. Authors should report the full distribution, time-dependent changes, and orthogonal size measurements rather than presenting a single threshold as proof of translational suitability.

### 4.4. Material Composition, Luminal Protection, and Triggered Release

Material choice determines payload compatibility, degradation products, colloidal stability, immune recognition, and manufacturing feasibility. Chitosan and thiolated chitosan can increase mucoadhesion and epithelial contact [84,85,86], whereas alginate–chitosan architecture can reverse the exposed charge and improve mucus penetration [36]. A silk-fibroin system stabilized with poloxamer 407 produced approximately 170-nm, narrowly dispersed, negatively charged resveratrol nanoparticles with improved mucus penetration and efficacy in experimental colitis [87]; this supports the combined importance of core material and exposed hydrophilic layer, while remaining a formulation-specific animal result. Lipid particles and biomimetic carriers offer membrane compatibility and cellular interaction, but their composition, oxidation, digestion, and immune effects must be controlled [5,58,59,88,89]. Edible plant-derived vesicles show biological activity in colitis models [13,14], yet batch definition, cargo variability, dose normalization, and scale-up remain distinct translational challenges. Recent bio-derived platforms also illustrate why carrier activity, and delivery function must be distinguished. Li et al. investigated exosome-like nanovesicles from *Rosa roxburghii* and *Artemisia sphaerocephala*. Their in vitro and in vivo findings linked barrier protection to increased mucus secretion, tight-junction protein expression, and anti-inflammatory macrophage polarization, with additional microbiota modulation reported for *R. roxburghii* vesicles [90]. Such naturally derived vesicles should be distinguished from engineered membrane-coated biomimetic carriers, although both approaches exploit biological components [5]. For either platform, improved inflammatory outcomes may reflect intrinsic material or endogenous cargo activity rather than selective delivery of an added drug. Clinical efficacy remains unestablished.

Protection from gastric acid and premature release is not a preprocessing detail; it determines which formulation reaches the target. Enteric polymers, hydrogels, multilayers, and hybrid lipid–polymer structures can delay release until intestinal or colonic conditions. In mouse IBD models, pH-sensitive coating increased the diameter of budesonide PLGA particles from approximately 200 to 240 nm, reduced acidic release, and improved outcomes compared with plain nanoparticles and free drug at the same dose [39]. Thus, a modest size change cannot be interpreted apart from the coating’s dominant effect on release location.

The proposed framework explicitly accommodates gastric-labile nanoparticles enclosed in a pH-sensitive microcapsule or microparticle that protects them during gastric transit and releases them in the intended intestinal region. Gastric stability is therefore a requirement of the administered dosage form, not necessarily of the isolated nanoparticle. As a preclinical example, Naeem et al. encapsulated budesonide-loaded PLGA nanoparticles in Eudragit FS30D microparticles; the hybrid system limited premature drug release at gastric and proximal-intestinal test pH and improved inflammatory outcomes relative to nanoparticles alone in a mouse colitis model [91]. This supports staged protection and release but does not establish clinical efficacy or universal performance across IBD phenotypes.

For such hierarchical systems, we propose sequential testing of the complete dosage form through gastric and intestinal conditions, followed by characterization of the liberated nanoparticles and payload. Relevant endpoints include premature leakage, protection against acid and enzymes, microcapsule dissolution and nanoparticle liberation, post-release size distribution and aggregation, payload retention or intended release, and retained biological activity. Microcapsule dissolution, nanoparticle release, and drug release should be measured separately. The release trigger must be matched to the intended small-intestinal or colonic site and tested across relevant pH and transit-time ranges rather than assumed to operate only in the intestine under all conditions. Enteric release establishes a regional-delivery strategy; inflammation selectivity still requires matched healthy and inflamed comparisons after release.

Inflammation-responsive systems use ROS, enzymes, pH, or disease-associated ligands to trigger release or retention [4,52,53,54,55,56,57]. Their selectivity must be tested against the relevant physiological range. For ROS-responsive carriers, release profiles should include healthy and inflamed ROS concentrations, antioxidant-rich media, and the possibility that carrier degradation consumes or generates reactive species. For pH-responsive systems, regional variability, food effects, and active inflammation should be considered. A responsive material is mechanistically selective only if differential activation is measured under paired conditions and linked to released payload and function.

## 5. From Accumulation to Productive Delivery

Bulk tissue fluorescence provides evidence of label-associated exposure but alone does not establish intact-carrier localization or productive delivery. It may reflect intact particles, free dye, degraded fragments, adsorbed protein, luminal residue, or uptake by different cell types. Similarly, an anti-inflammatory effect may arise from the payload, carrier material, altered microbiota, reduced systemic exposure, or nonspecific lesion trapping. A strong study therefore separates the formulation question into four sequential claims: localization, selectivity, payload delivery or target engagement, and safety.

Localization asks where carrier components and payload are found. Microscopy can map their anatomical distribution across lumen, mucus, epithelium, lamina propria, and immune cells; identifying intact carriers additionally requires material-specific characterization and label-stability controls. Selectivity requires a paired healthy comparator at the same dose and interval; comparison with an untreated inflamed group does not establish diseased-tissue targeting. Productive delivery requires released payload, intracellular bioavailability, target engagement, or a pharmacodynamic effect. Safety requires epithelial and immune readouts in the barrier state where the carrier is expected to act.

No universally validated method can be assumed to distinguish intact nanoparticles from released fluorophores or fluorescent degradation products with 100% accuracy throughout deep ex vivo intestinal tissue. The studies below demonstrate formulation-specific analytical approaches, not a validated gold standard for human IBD tissue [92,93,94]. Covalent attachment does not by itself resolve the problem: Roussel et al. combined fluorescent and radioactive labels in polymeric nanoparticles and showed that degradation of the polymer anchor could release fluorescent species and distort biodistribution estimates [92]. Thus, an enduring fluorescent signal may represent a labelled fragment rather than an intact carrier.

Förster resonance energy transfer (FRET) provides a proximity-sensitive readout beyond single-channel intensity. Bouchaala et al. used calibrated near-infrared ratiometric FRET to estimate lipid-nanocarrier integrity in mouse blood, liver, and tumor [93]. Long et al. combined FRET with particle characterization to distinguish stages of lipid-nanoparticle dissociation and followed disassembly in cultured human corneal epithelial cells [94]. Neither study establishes deep-tissue accuracy in inflamed human intestine. FRET reports donor–acceptor proximity, not complete structural or chemical integrity; partial disruption with retained proximity need not eliminate the signal. Loss of FRET therefore requires interpretation against intact and disrupted formulation controls, rather than being equated automatically with complete degradation. Likewise, overlap of two fluorescence channels is supportive but cannot alone establish that both labels reside within one intact particle.

For deep ex vivo tissue, depth-dependent attenuation, background fluorescence, and unequal transmission of donor and acceptor emission complicate quantitative interpretation [93]. We therefore propose matrix- and depth-matched controls containing intact particles, deliberately disrupted particles, free dye, and relevant labelled degradation products, together with label-stability testing under the exposure and sample-processing conditions. Sectioning can improve anatomical access but does not establish chemical identity. Orthogonal characterization may include material-appropriate electron microscopy or analysis of recovered material: Roussel et al. used size-exclusion separation with fluorescence and radioactivity detection to distinguish nanoparticle-associated signals from smaller degradation products in vitro [92]. Applying such separation to tissue extracts would require validation of recovery and processing-induced disassembly and would sacrifice spatial information. Reports should state the sampled depth, detection limits, recovery, and residual uncertainty. Where integrity remains unresolved, the defensible endpoint is tissue-associated label rather than confirmed intact-carrier delivery; payload release or target engagement must still be measured separately.

Therapeutic outcome alone cannot determine which formulation property was responsible. In animal studies, body weight, disease activity index, colon length, histology, cytokines, and oxidative markers are valuable, but they do not replace exposure measurements. Conversely, improved exposure without functional benefit may reveal poor release, intracellular trapping, inadequate potency, or toxicity. Section 3.2 illustrates this distinction using the si*JAK1* model [37].

Dose matching is essential. Comparing a nanoparticle at one payload dose with free drug at another, or using different dosing schedules, prevents attribution of benefit to the carrier. Studies should report payload dose, carrier mass, encapsulation efficiency, free-payload fraction, release before dosing, administered volume, fed state, and recovery. When a carrier has intrinsic anti-inflammatory activity, an empty-carrier control is required. For targeted formulations, an untargeted composition-matched control and a receptor-blocking or competition experiment greatly strengthen causal inference.

The disease features described in Section 3.2, Section 3.3, Section 3.4 and Section 3.5 also constrain model applicability. Acute chemical colitis is useful for screening but may exaggerate erosions and passive retention. A formulation designed for superficial ulcerative colitis lesions should not be generalized to stricturing or transmural Crohn’s disease without appropriate models and tissue. Clinical heterogeneity also extends beyond intestinal disease phenotype. In a single-center retrospective cohort of 7869 patients, Huang et al. found that diabetes was independently associated with classification as severe IBD (odds ratio 3.81, 95% confidence interval 1.85–7.98) [95]. This study did not evaluate mucosal barrier properties, nanocarrier disposition, or drug-delivery performance, and its observational design does not establish causality. It nevertheless illustrates why metabolic comorbidities and systemic disease state should be documented in translational studies and why formulation findings should not be generalized across clinically dissimilar IBD populations.

The same discipline is needed when comparing targeting strategies. Historical comparisons of passive size-dependent deposition, charge-tunable carriers, and inflammation-specific ligands show that several routes can increase colonic exposure [43,96,97,98]. Recent systematic and scoping syntheses confirm the breadth of candidate platforms but also the heterogeneity of models and outcome definitions [99]. A higher signal than free drug or an untreated control is insufficient to rank these strategies. Head-to-head comparisons require the controls outlined above and consistent localization and functional readouts.

## 6. Barrier-State-Matched Translational Benchmarking

### 6.1. Model Selection Should Follow the Formulation Claim

No single model reproduces the full oral route. Acellular media and mucus assays define colloidal identity, diffusion, and release but cannot establish epithelial or immune function. Caco-2 monolayers provide a reproducible permeability screen but do not reproduce native mucus, immune sampling, regional epithelium, or ulcer heterogeneity. Mucus-producing co-cultures add an important barrier component, while three-cell inflamed systems allow epithelial–immune interaction and safety testing [25,37,38,69]. The model should be selected because it can falsify a claim, not because it is the most complex available.

Organoids and organoid-derived monolayers preserve patient- and disease-associated epithelial features better than immortalized lines [100,101]. Their limitations include inward-facing lumens in three-dimensional structures, variable mucus, incomplete immune and vascular components, and difficult dose normalization. Patient-derived organoids can nevertheless reveal payload function in diseased epithelium; nanoencapsulated β-carotene, for example, produced stronger anti-inflammatory effects than free compound in UC-derived organoids [32]. This result supports epithelial activity, not oral bioavailability or lesion targeting.

Ex vivo human mucosa and biopsies offer high translational relevance for short-term localization and transport [27,31,64]. They also introduce donor variability, regional heterogeneity, prior medication, limited viability, and low throughput. Paired inflamed and macroscopically non-inflamed tissue from the same donor can reduce between-patient variability. Clinical metadata and histological confirmation are needed to interpret results. Human tissue should be used to test a focused mechanism after formulation screening, not as a substitute for basic physicochemical characterization.

Animal models integrate transit, distribution, immune response, and efficacy but differ from human IBD in lesion architecture, mucus, microbiota, dose scaling, and disease time course. Acute dextran sulfate sodium colitis is particularly useful for testing lesion-associated retention and epithelial injury, yet it may favor particles that exploit severe erosions. Confirmation in a second model or human tissue is appropriate when the proposed mechanism depends on chronic remodeling, immune targeting, or geometry-specific uptake.

### 6.2. Proposed Reporting Domains and Candidate Advancement Criteria

**Figure 2:**
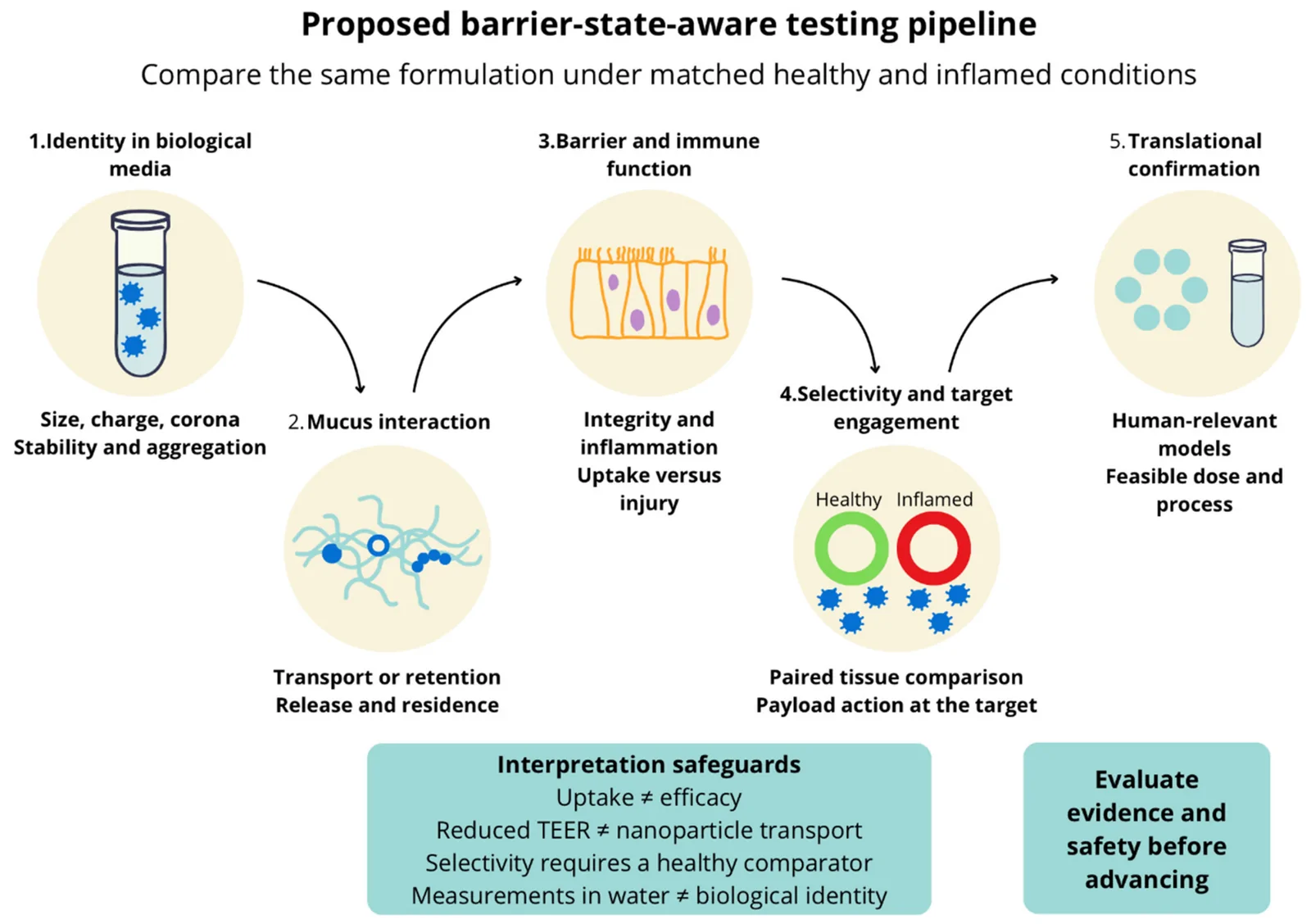
Proposed barrier-state-aware testing pipeline. The proposed sequence comprises five linked stages: (1) characterization of nanocarrier identity in biological media; (2) assessment of mucus transport or retention, release, and residence; (3) evaluation of barrier integrity, immune responses, and injury alongside uptake; (4) paired healthy–inflamed tissue comparison with assessment of selectivity and payload target engagement; and (5) confirmation in human-relevant models together with evaluation of dose and process feasibility. Evidence and safety should be assessed before advancement using prespecified, task-specific criteria. The safeguards highlight that uptake alone does not establish efficacy, reduced transepithelial electrical resistance (TEER) does not demonstrate nanoparticle transport, inflammation selectivity requires an appropriate healthy comparator, and measurements in water do not define biological identity. Arrows indicate a proposed testing sequence, not guaranteed progression. Icons are schematic, and this framework requires validation rather than constituting an established regulatory standard. The circular panels represent successive stages of the proposed testing pipeline, and the connecting arrows indicate progression between stages. Light-blue wavy lines represent mucus, whereas the green and red rings in Step 4 denote healthy and inflamed conditions, respectively. The remaining colors and symbol sizes are used for visual clarity and have no quantitative meaning.

Figure 2 organizes the proposed assessments into a staged testing pipeline; its predictive value remains unvalidated. Tracking the same batch across stages and comparing healthy and inflamed conditions at matched formulation, payload dose, and sampling time can improve interpretability. As a proposed decision checkpoint, unexplained aggregation or loss of payload in relevant media should prompt characterization of the exposed species and reassessment before interpreting uptake. Intended disassembly or reproducible transformation is not, by itself, a reason to reject a formulation. Accordingly, gastric lability of an isolated nanoparticle is not an exclusion criterion when a validated protective dosage form preserves the intended delivery function; its protection and subsequent release should be assessed as described in Section 4.4.

We propose the following reporting domains, with measurements selected according to the intended mechanism and development stage rather than treated as a mandatory checklist. Physicochemical characterization can include composition, morphology, hydrodynamic size distribution, PDI, zeta potential in relevant media, encapsulation efficiency, free-payload fraction, release kinetics, and storage and post-exposure stability. Mucus assessment can include source and disease state, diffusion or retention, washout, and payload release. Epithelial–immune assessment can include TEER, paracellular flux, junctional imaging, cell-specific uptake, cytokines, oxidative stress, and viability or barrier recovery. Localization assessment can include carrier and payload mapping, with intact-carrier measurements when carrier persistence is part of the claim and biodistribution when systemic exposure is relevant.

Candidate endpoints differ by delivery objective. For a mucus-penetrating system, candidate endpoints include reproducible behavior in intestinal media, improved diffusion over an appropriate comparator, retained payload activity, and acceptable epithelial tolerability. For a lesion-retentive system, they include preferential localization when inflammation selectivity is claimed, sustained local payload availability, and systemic exposure compatible with the intended use. For immune-cell delivery, they include cell-type localization, functional target engagement, and acceptable effects on barrier and immune function. Acceptance limits should be prespecified with reference to assay variability, comparator performance, exposure, and the intended benefit–risk profile; this review cannot supply validated numerical cutoffs. A proposed validation strategy is to test whether these criteria discriminate functional delivery and tolerability across independently manufactured batches, relevant models, and donor samples, followed by clinical evaluation where justified. Neither passing a checkpoint nor failing one model determines clinical suitability.

The provenance of these candidate criteria is mixed and endpoint-specific. Characterization in biological media draws on analytical guidance and experimental transformation studies [24,50,75,76,77,78], not clinical advancement thresholds. Mucus-penetration criteria are motivated mainly by ex vivo mucus and preclinical transport studies [36,40,41,42,44]; study [44] includes human cervicovaginal mucus, not human intestinal disease. Lesion-retention criteria draw on animal localization/biodistribution [26,28,29], a rectal human mechanistic study [27], and ex vivo human intestinal tissue [31], without demonstrating benefit after oral dosing in patients. Immune-cell delivery and barrier-safety criteria draw on in vitro co-cultures [37,38], patient-derived organoids [32], ex vivo human tissue [31], and animal experiments [88,89]. Clinical trials provide product-specific outcome evidence [33,34,35], but none validates this combined decision sequence. The model-specific questions, suggested readouts, and principal limitations underlying this benchmarking approach are summarized in Table 3.

**Table 3:** Proposed model–question–readout matrix for translational benchmarking.

Model	Primary Question	Suggested Readouts	Principal Limitation
Sequential saliva/gastric/intestinal media	How does the formulation transform, and is that transformation compatible with its intended function?	Size distribution, PDI, zeta potential, aggregation, corona/ligand accessibility, payload recovery and release	No mucus, epithelium, clearance, or disease heterogeneity.
Native or disease-relevant mucus/ex vivo mucus	Does the carrier penetrate, remain, or release as intended?	Single-particle tracking or validated diffusion method, spatial distribution, washout, mucin binding, payload release	Source, storage, dilution, and architecture strongly affect results.
Caco-2 or epithelial monolayer	Does the carrier alter epithelial integrity or cross a controlled barrier?	TEER, size-matched tracers, tight-junction imaging, mass balance, intact-carrier/payload quantification	Limited mucus and immune relevance; reduced TEER can create artefactual transport.
Mucus-producing or epithelial–immune co-culture	How does inflammation change transport, uptake, and safety?	TEER/flux, mucus distribution, cell-specific uptake, cytokines, oxidative stress, barrier recovery	Simplified immune repertoire and lesion architecture; induction protocol must be reported.
Patient-derived organoid/monolayer	Is payload function retained in disease-relevant epithelium?	Target engagement, cytokines, viability, differentiation, patient-stratified response	Luminal dosing, mucus, flow, and immune components often incomplete.
Ex vivo human inflamed and paired non-inflamed tissue	Is localization selective in human lesions?	Spatial imaging, tissue recovery, cell-type colocalization, payload release, short-term target engagement	Low throughput, donor variability, short viability, medication and regional confounding.
Animal colitis model	Do transit, exposure, efficacy, and safety align in vivo?	GI distribution, lesion localization, systemic organs, pharmacokinetics, histology, barrier function, immune response	Model-specific erosions and acute inflammation may not generalize to chronic human IBD.
Clinical study	Does a defined formulation improve patient-relevant outcomes?	Disease activity, endoscopy/histology, pharmacokinetics, safety, formulation quality and adherence	Efficacy alone does not prove barrier-state-aware targeting without exposure/localization evidence.

### 6.3. Making Negative Preclinical Findings Publishable and Reusable

Negative findings can inform oral IBD formulation development when they identify why a carrier fails under defined barrier conditions. Reports should document composition and batch, disease state, dose, exposure, release or target engagement, safety, and assay responsiveness to distinguish delivery failure from inadequate payload activity or model limitations. Prespecified outcomes, justified sample sizes, appropriate controls, and effect estimates with uncertainty are essential; ARRIVE 2.0 supports transparent reporting of animal experiments [102]. Statistical nonsignificance alone does not establish ineffectiveness. Reporting unsuccessful formulations and adverse effects alongside positive findings can define the limits of a delivery strategy without implying universal failure.

Publication incentives can be addressed through Registered Reports, with in-principle acceptance based on the design and conditional on protocol adherence and quality checks rather than a positive outcome [103], and journals with outcome-independent criteria, such as PLOS ONE [104]. For nanocarrier studies, concise reports of well-characterized failure mechanisms or replication results, linked to accessible protocols, datasets, and analysis code, would support comparison across formulations and barrier states. Funders and institutions could recognize complete reporting irrespective of outcome. These approaches may improve the availability and reuse of negative evidence, but neither preregistration nor repository deposition guarantees journal acceptance or substitutes for peer review.

## 7. Clinical Translation: Clinical Evidence, Manufacturing, and Regulatory Challenges

### 7.1. What the Clinical Trials Establish—And What Remains Unresolved

The oral trials below assess product-specific therapeutic activity in UC. They do not directly establish preferential delivery to inflamed intestine or isolate the contribution of nanoscale formulation [33,34,35].

Masoodi et al. randomized 56 patients to nanomicellar curcuminoids (80 mg three times daily) or placebo, both alongside mesalamine, for four weeks. The endpoint SCCAI was 1.71 ± 1.84 versus 2.68 ± 2.09 (*p* = 0.050), with improvements in selected symptoms [34]. This finding supports further investigation of adjunctive treatment, but the small sample, short observation period, and borderline between-group SCCAI result limit conclusions about durable disease control. Without a conventional-curcumin comparator, the trial cannot establish whether nanomicellization itself provides additional clinical benefit.

Ahamed et al. evaluated oral nano-vitamin D at 60,000 IU daily for eight days in patients with active UC and serum vitamin D below 40 ng/mL. At four weeks, a three-point UCDAI response occurred in 53% of treated patients versus 13% of controls (*p* = 0.001) [35]. However, this was a clinical-response endpoint rather than evidence of sustained remission. Correction of low vitamin D status provides an alternative explanation to intestinal targeting, and the absence of a conventional-vitamin D arm prevents attribution of benefit specifically to the nanoformulation.

Daryani et al. studied 43 patients with mild-to-moderate UC receiving nano-curcumin or placebo alongside 5-aminosalicylic acid for one month. Clinical and endoscopic responses favored nano-curcumin (*p* = 0.031 and *p* = 0.005, respectively), whereas between-group differences in inflammatory markers, total antioxidant capacity, and fecal calprotectin were not significant [33]. This mixed endpoint pattern warrants confirmation in larger studies rather than interpretation as uniform suppression of intestinal inflammation.

Collectively, these trials support further clinical evaluation but do not establish maintenance efficacy, long-term safety, superiority over corresponding non-nano formulations, or applicability to Crohn’s disease. Their different payloads, doses, and endpoints also preclude ranking nanocarrier platforms [33,34,35].

### 7.2. Designing Clinically Informative Translational Studies

Clinical development should distinguish induction from maintenance treatment and use prespecified outcomes that capture both symptoms and endoscopic disease activity. The FDA’s 2022 draft guidance for UC drug development provides a disease-specific framework for trial populations, efficacy endpoints, and safety assessment [105]. For oral nanomedicines, we additionally propose reporting formulation composition, administered carrier and drug doses, concomitant therapy, adherence, disease location, and baseline inflammatory activity. Effect sizes and confidence intervals should accompany significance tests.

Mechanistic substudies should be selected according to the proposed delivery mechanism; we do not propose a universal tracker that is established as safe in humans and proves the integrity of all biodegradable carriers. Detection of a lipid, sugar, polymer fragment, or attached label in a biopsy does not establish persistence of the assembled nanoparticle. Components may enter endogenous metabolic pools after degradation, and even an intact polymer chain need not remain assembled into a particle. After complete degradation, residual labels cannot retrospectively prove intact-carrier transport. Label stability and carrier integrity must therefore be distinguished [92].

A concrete human imaging precedent is copper-64-labelled MM-302, an intravenously administered liposomal formulation studied by PET in patients with metastatic breast cancer [106]. This demonstrates formulation-specific clinical imaging feasibility, not a universal integrity assay for oral lipid or polysaccharide carriers. Radiotracer detection alone cannot distinguish an intact assembly from released or metabolized label. The dual-labelling and FRET approaches discussed in Section 5 remain formulation-dependent research methods [92,93,94], not established universal trackers for human intestinal biopsies. Any proposed labelled clinical formulation requires assessment of label retention, altered formulation behavior, tracer toxicology, and, for radionuclides, radiation dosimetry.

Where intact-carrier transport is central to the claim, we propose a material-specific combination of chemical identification, size- or structure-sensitive analysis, and spatial localization, validated in the relevant tissue matrix. Chemical detection or colocalization alone is insufficient. Analysis of recovered material must address endogenous background, extraction recovery, processing-induced disassembly, and detection limits; Section 5 details these analytical limitations. If no suitable assay is available, the study should report carrier-component exposure or label localization without claiming confirmed intact-carrier delivery.

Biopsy sampling should be confined to ethically justified mechanistic substudies with informed consent, preferably coordinated with clinically indicated endoscopy where feasible. For carriers designed to degrade and release drug, more relevant endpoints may be local payload exposure, target engagement, pharmacodynamic response, and safety. Total, free, and nanomaterial-associated drug should be distinguished where feasible using validated methods, consistent with FDA guidance [107]. These endpoints do not prove carrier integrity but can test productive delivery without imposing routine biopsies or persistent-carrier detection on every nano-enabled product.

### 7.3. Regulatory and Safety Considerations

The FDA guidance on drug products containing nanomaterials emphasizes product-specific characterization and assessment of how nanoscale attributes affect quality, exposure, and safety [107]. Familiarity with an active ingredient or excipient does not by itself establish the safety of its reformulated product. Relevant questions include altered absorption, local gastrointestinal effects, carrier degradation, and possible accumulation of poorly soluble or persistent materials. For chronic IBD treatment, repeated-dose exposure therefore requires particular attention.

A further challenge is bridging the material used in exploratory studies to that manufactured for clinical use. Changes in process, scale, or manufacturing site may alter product performance and require analytical, nonclinical, or clinical bridging, depending on their impact [107]. For locally acting products, similar plasma exposure may not establish equivalent intestinal delivery.

The regulatory route should be discussed with the relevant authority during development and determined for the specific finished product, rather than for a generic carrier platform. In the United States, a 505(b)(2) application may permit reliance on existing findings for a listed drug when an adequate scientific bridge can be established. However, comparable plasma exposure alone may be insufficient for nanomaterial-containing products, and additional nonclinical or clinical evidence may be needed [107]. Barrier-state-aware testing therefore supplements, rather than replaces, the evidence supporting quality, safety, and efficacy for the intended indication.

### 7.4. Manufacturing Scalability, Reproducibility, and Quality Control

Translation also requires reproducible manufacture at a clinically feasible dose and cost. Following ICH Q8(R2), development should identify material attributes and process parameters that influence product quality [108]. Applied to oral nanocarriers, relevant variables may include raw-material composition, mixing conditions, solvent removal, coating, and drying. Their importance must be established experimentally rather than inferred from successful preparation of a laboratory batch. We propose comparing independently manufactured batches across development stages; repeated measurements of one batch demonstrate analytical precision, not manufacturing reproducibility.

A product-specific control strategy should connect critical quality attributes with intended performance [108]. Candidate measurements include drug content and uniformity, size distribution, morphology, free versus associated drug, surface functionality, impurities, and discriminatory release testing [107,108]. Functional assays should be selected and validated for the particular formulation. Biorelevant gastrointestinal testing can support development but does not automatically constitute a validated routine batch-release method.

Stability studies should use the intended packaging and appropriate long-term and accelerated conditions, with methods capable of detecting changes relevant to quality, safety, and efficacy [109]. For a nanocarrier, we propose monitoring not only drug degradation but also physical instability, premature release, and, where applicable, performance after reconstitution. Finally, gastrointestinal transformation should be reproducible and compatible with the delivery mechanism: a carrier designed to disassemble and release its payload need not remain structurally intact throughout transit. Shelf life must be supported by data for the marketed dosage form and packaging; intestinal responsiveness does not itself imply poor storage stability. A dry or enteric-protected presentation is a candidate strategy only if post-storage release and, where relevant, redispersion remain acceptable [109]. 

The human clinical and mechanistic evidence relevant to the subsequent translational discussion is summarized in Table 4.

**Table 4:** Human clinical and mechanistic evidence relevant to nano-enabled interventions in IBD.

Study	Design	Intervention	Main Finding	What the Study Does Not Establish
Masoodi et al. [34]	Human clinical: Randomized double-blind trial; *n* = 56; UC	Nanomicellar curcuminoids 80 mg three times daily + mesalamine vs. placebo + mesalamine; 4 weeks	SCCAI 1.71 ± 1.84 vs. 2.68 ± 2.09 (*p* = 0.050); selected symptom improvements	Short duration; no formulation localization or exposure mechanism; adjunctive regimen.
Ahamed et al. [35]	Human clinical: Double-blind randomized placebo-controlled trial; active UC	Nano-vitamin D 60,000 IU/day for 8 days	Three-point UCDAI response at 4 weeks: 53% vs. 13% (*p* = 0.001)	Not designed for intestinal lesion targeting; mechanism may reflect systemic vitamin D exposure.
Daryani et al. [33]	Human clinical: Randomized placebo-controlled double-blind trial; *n* = 43; mild-to-moderate UC	Nano-curcumin 80 mg twice daily vs. placebo, alongside 5-aminosalicylic acid; 1 month	Clinical and endoscopic responses favored nano-curcumin (*p* = 0.031 and *p* = 0.005); no significant between-group differences in inflammatory markers, total antioxidant capacity, or fecal calprotectin	Short-term adjunctive efficacy does not establish durable remission, superiority over conventional curcumin, or inflammation-selective delivery.
Schmidt et al. [27]	Human in vivo mechanistic study; 33 IBD patients and 6 controls; not a therapeutic trial	Rectal 250 nm NPs and 3.0 µm MPs	MPs accumulated in ulcerated lesions; NPs only traces	Direct human localization evidence after rectal dosing; not an oral therapeutic trial or validation of a preferred particle size.

### 7.5. Integration with QbD, Target Product Profiles, and Translational Development Strategies

The proposed barrier-state-aware framework is a disease-specific application of established development principles, rather than an alternative to Quality-by-Design (QbD) or target product profile (TPP) planning. A TPP defines the intended use, target population, and desired product characteristics, including efficacy and safety [110]. For an oral IBD therapy, we propose specifying disease phenotype and location, induction versus maintenance use, intended site of action, and acceptable dosing burden before selecting a carrier platform. Barrier-state-aware benchmarking then translates these objectives into biological conditions under which delivery performance should be tested.

The TPP should be distinguished from the quality target product profile (QTPP), which describes the quality characteristics needed to support the intended product’s safety and efficacy. Within QbD, material attributes and process parameters are linked to critical quality attributes through risk assessment and experimental understanding [108]. The present framework complements this approach by asking under which intestinal conditions a candidate attribute predicts useful delivery. Inflammation is not itself a manufacturing parameter or product quality attribute; it is a biological condition against which formulation performance can be evaluated. Thus, mucus composition or epithelial injury can be incorporated into development studies alongside formulation variables without being misclassified as components of a manufacturing design space.

Established translational nanomedicine strategies similarly integrate physicochemical characterization with biological compatibility, pharmacology, and toxicology. For example, the National Cancer Institute’s Nanotechnology Characterization Laboratory uses a standardized assay cascade with research plans tailored to the formulation, indication, dose, and administration route [111]. Although developed in an oncology setting, this provides a useful organizational comparison. Our framework retains that staged, product-specific logic but focuses on gastrointestinal transformation, mucus interactions, and matched intestinal barrier states. It does not replace conventional pharmacokinetic, toxicological, manufacturing, or clinical assessment.

As an illustrative development scenario, consider two coatings for a locally acting oral UC formulation. The TPP would define the intended clinical benefit and patient population, while the QTPP would specify relevant dosage-form, release, and stability characteristics. QbD studies would examine how coating composition and processing affect reproducible product quality. Barrier-state-aware experiments would then compare the coatings at equal payload doses under defined inflamed and non-inflamed conditions, measuring local payload availability, target engagement, and epithelial injury. A coating that increases tissue-associated fluorescence without improving functional delivery would not justify a targeting advantage. Conversely, a simpler formulation achieving comparable functional benefit with fewer manufacturing steps could be preferred. This example illustrates a decision process, not a clinically validated selection rule.

Commercially, we envisage a standardized product for a defined indication and patient population, not bespoke manufacture for each patient or reformulation whenever inflammation fluctuates. For example, a fixed-composition oral product developed for induction therapy in colonic UC could be tested across the relevant range of mucus properties, pH, and inflammation severity. This is an illustrative development scenario, not a validated product recommendation. A common manufacturing platform with a limited number of justified variants could support scalability; however, shared platform chemistry would not automatically establish equivalent performance or safety across variants.

A practical development sequence would therefore define the target population and intended clinical advantage, select a reproducible formulation and dosage form, establish the process controls and shelf life described in Section 7.4, and evaluate clinical benefit and safety in that population. Patient-selection criteria should be prespecified and clinically justified, while any proposed molecular biomarker for treatment selection would require prospective validation. The target product profile should also address dosing burden and storage conditions [110]. Biological robustness across the intended population is the development goal; a narrowly tuned formulation that fails as barrier conditions change would be a liability, not a desired form of precision delivery.

Commercial viability remains unproven by this framework. We propose assessing manufacturing yield, raw-material supply, batch-release testing, storage and distribution costs, the size of the eligible population, and added benefit over available therapy early in development. The additional cost of specialized delivery would need to be justified by a meaningful improvement in efficacy, safety, adherence, or dosing burden. If such an advantage is not demonstrated, a simpler formulation should be preferred. Thus, barrier-state-aware development is intended to guide selection and de-risk a scalable product, not to exempt specialized systems from commercial or regulatory constraints. Validation requirements are detailed in Section 6.2.

### 7.6. Future Perspectives: AI-Assisted Optimization and Molecularly Informed Precision Delivery

Artificial intelligence (AI) and machine learning may help prioritize formulations and interpret barrier interactions, but direct evidence in oral IBD nanomedicine remains limited. Published examples include AI-guided ionizable-lipid discovery for mRNA delivery [112] and prediction of nanoparticle mobility in human airway mucus [113]; neither validates formulation selection in inflamed intestine. Application to IBD would require standardized datasets linking composition and post-exposure properties to defined intestinal barrier states, functional delivery, and safety, including unsuccessful formulations. Predictions should be tested on independent donors and manufacturing batches, with uncertainty reported, rather than optimized for uptake alone.

Gastrointestinal digital twins remain a longer-term research direction. Existing simulations of gastric fluid dynamics, tablet motion, and dissolution [114] are not longitudinally updated patient-specific models of oral nanocarrier delivery. Their nearer-term value is hypothesis testing and sensitivity analysis, not clinical formulation selection. Such selection would require validated representation of regional transit, carrier transformation, and release, together with independent exposure measurements and prospective evaluation as disease activity changes.

Spatial transcriptomics and multi-omics could provide complementary information for defining relevant biological states. Li et al. constructed a spatial transcriptomics atlas using intestinal tissues from controls and patients with UC or Crohn’s disease [115]. Separately, longitudinal multi-omics profiling by Lloyd-Price et al. documented disease-associated changes in microbial activity and metabolite pools [73]. These resources could inform hypotheses about target-cell location, local inflammatory programs, and environments affecting carrier transformation. Nevertheless, transcript abundance does not establish accessible surface-protein expression, and stool omics cannot directly define conditions at a mucosal lesion. Candidate targeting ligands or release mechanisms would therefore require protein-level, spatial, and functional verification in relevant tissue.

A practical route toward precision oral drug delivery would connect molecularly defined patient subgroups with a limited set of reproducible formulation options. As a hypothetical example, a spatially identified inflammatory cell population could motivate ligand selection, while matched tissue assays determine whether the ligand actually improves payload delivery without increasing injury. Microbial or metabolite signatures could similarly guide testing of release robustness but should not be treated as validated formulation-selection biomarkers. The decisive endpoint would be prospective evidence that biomarker-informed selection improves outcomes over an appropriate non-stratified strategy.

## 8. Conclusions

Comparative studies indicate context-dependent differences in performance, but heterogeneity in surface chemistry, geometry, route, lesion architecture, and endpoints limits cross-study rankings. Human observations provide important but restricted support: a rectal-administration study found preferential accumulation of microparticles in ulcerated lesions [27], while an ex vivo tissue comparison found selective uptake of wormlike micelles not reproduced by the other tested shapes [31]. Neither establishes an optimal oral formulation or clinical superiority.

We therefore propose three evidence-informed research principles rather than validated design rules. First, define the task—luminal protection, mucus retention, mucus penetration, epithelial access, immune-cell delivery, or triggered release—before optimizing a property; this is an author-proposed synthesis of the model-specific comparisons in Table 2. Second, characterize carrier transformation in relevant media and use matched healthy and inflamed comparators when testing inflammation selectivity, informed by mechanistic experiments, animal comparisons, and limited human localization evidence [24,26,27,28,31]. Third, separate localization from function: tissue-associated signal, reduced TEER, or cellular uptake does not prove intact-particle transport, productive payload release, target engagement, or safety; the in vitro co-culture findings [37,38] and the endpoint limitations of human clinical trials [33,34,35] illustrate why these claims require separate measurements.

The next step is prospective validation of the proposed pipeline (Figure 2): whether its criteria improve formulation selection and predict functional delivery and tolerability across independent batches, models, and donor samples, followed by clinical evaluation where justified.

## Figures and Tables

**Table 1:** Primary comparative evidence that constrains formulation claims.

Study	Model/Comparator	Formulation Variable	Key Observation	Defensible Interpretation
Lamprecht et al. [26]	Animal in vivo: rat colitis vs. healthy colon	0.1, 1, and 10 µm polymeric carriers	Colonic deposition: 14.5 vs. 2.2%; 9.1 vs. 2.0%; 5.2 vs. 1.4%, respectively	Supports inflammation-dependent deposition in this model; does not localize signal within the lesion.
Schmidt et al. [27]	Human in vivo mechanistic: 33 IBD patients; 6 controls; rectal dosing	250 nm NPs and 3.0 µm MPs; rectal dosing	3 µm MPs enriched in ulcerated lesions (covered area 1.28%, range 0.83–3.45); NPs only traces	Human result contradicts a universal small-particle advantage; suggests lesion discontinuities/persorption.
Wachsmann et al. [29]	Animal in vivo: murine experimental colitis	≈200 nm particles with different surfactants	Polysorbate-20 formulation showed 34.8-fold greater inflamed/healthy colonic content and 4.5-fold greater content than SDS particles	Supports a surface-chemistry effect within the tested murine system, not a universal ranking over size.
Schmid et al. [28]	Animal in vivo: healthy/colitic mice; ex vivo adhesion	PEG- vs. HA-functionalized mesoporous silica NPs	Inflammation favored HA retention; biodistribution differed, with organ signal especially for PEG in colitis	Supports assessing retention alongside systemic redistribution; findings remain model- and material-specific.
Vafaei et al. [30]	In vitro: inflamed vs. untreated Caco-2 cells; CD44-negative comparator	HA NPs, 177–293 nm, negative charge	Higher uptake in inflamed CD44-high Caco-2 cells; budesonide NPs reduced IL-8/TNF-α secretion more than free drug in vitro.	Links a disease-associated receptor and function, but cell model cannot establish human lesion selectivity.
Sun et al. [36]	Preclinical: mucus assays, ex vivo intestine, animal colitis model	Alginate–chitosan NPs, 257 nm, negative charge	2.9-fold higher mucus penetration than positive chitosan NPs; ex vivo retention >16 h	Charge effect is formulation- and assay-specific; penetration and retention can coexist.
Gardey et al. [31]	Ex vivo human: inflamed intestinal tissue and controls	Composition-related spheres, vesicles, wormlike micelles	Selective inflamed-tissue uptake and immune-cell localization for wormlike micelles; not reproduced by other shapes	Ex vivo human evidence of shape-associated localization; does not establish oral delivery, shape alone as the cause, or clinical superiority.
Hartwig et al. [37]	In vitro: human three-cell inflamed “leaky gut” model	si*JAK1* nanoparticles	Accumulation in leaky regions/immune cells, but target engagement only 10–16% vs. 86% with tofacitinib	Access and uptake do not establish productive intracellular delivery.
Susewind et al. [38]	In vitro: healthy vs. IL-1β-stimulated three-cell model	Multiple nanomaterials	Inflammation changed toxicity and IL-8 response	Safety ranking can change with barrier state.
Ali et al. [39]	Animal in vivo: mouse IBD models	Budesonide PLGA NPs: plain ≈ 200 nm; pH-coated ≈ 240 nm	Coating reduced acidic release and improved colonic outcome vs. plain NPs/free drug at matched dose	Luminal protection and regional release are integral formulation variables.
Ngew et al. [32]	In vitro, patient-derived: UC organoids	β-carotene zein/pectin/PEG NPs	DSS organoids: TNF-α reduced ≈ 70% and IL-8 ≈ 31%; stronger than free β-carotene	Patient-derived epithelium strengthens relevance but lacks luminal transport, immune, and dosing complexity.

**Table 2:** Proposed task-specific assessment matrix for formulation variables. The suggested measurements are author-proposed assessment options informed by the cited studies, not validated pass/fail criteria. Their relevance and acceptance limits depend on the formulation, intended mechanism, assay performance, and development stage. Evidence provenance refers to the cited observation, not validation of the entire recommended assay set. Human mucus from a non-intestinal site is indirect evidence for IBD; ex vivo studies are distinct from clinical trials.

Variable	Intended Task	Evidence Provenance and Representative Sources	Main Uncertainty or Risk	Suggested Measurements for Claim Assessment
Size/distribution	Mucus diffusion, lesion retention, cellular access	Animal in vivo [26]; human in vivo, rectal [27]; animal intestinal mucus ex vivo [40,41,42].	No universal IBD threshold; result depends on route, chemistry, lesion, and endpoint.	Report full distribution in biological media; spatially localize intact carrier; compare healthy/inflamed.
Surface charge	Retention vs. penetration; cell interaction	Animal in vivo [29,43]; mucus/ex vivo and animal experiments [36]. No clinical charge-selection validation.	Water zeta potential is not the exposure identity; cationic adhesion may increase trapping/toxicity.	Measure in saliva/GI/mucus media; test washout, penetration, cytokines, and epithelial injury.
PEG/hydrophilic layer	Reduce nonspecific mucin binding	Non-intestinal human mucus ex vivo and mouse in vivo [44]; mouse colitis [28]; reviews [45,46].	Dense coating may reduce target-cell uptake or ligand engagement; much evidence is non-IBD.	Report molecular weight/density/conformation proxy, ligand exposure, stability, and paired mucus transport.
HA/ligands	Inflamed-cell or lesion association	In vitro Caco-2 [30]; mouse in vivo/ex vivo [28]. No clinical ligand-selection validation.	Receptor expression is heterogeneous; ligand can be corona-masked; retention may coexist with systemic exposure.	Use blocked/untargeted controls, receptor perturbation, spatial colocalization, and biodistribution.
Geometry	Alignment, rotational diffusion, immune-cell interaction	Ex vivo human intestinal tissue [31]; preclinical mucus experiments [47]; computational gels [48].	Mechanistic support is model-dependent; direct human IBD evidence is limited and clinical validation is lacking.	Use composition- and size-matched shapes; quantify aspect ratio/flexibility; validate in inflamed human tissue.
PDI	Batch quality and mechanistic interpretability	Analytical/methodological reviews [49,50,51]; not human IBD efficacy studies.	PDI <0.2 is a convention, not an IBD efficacy threshold.	Report distributions and time-course after media exposure; use an orthogonal sizing method.
pH/ROS/enzyme response	Regional or inflammation-triggered release	Preclinical formulation/release and animal colitis studies [39,52,53,54,55,56,57]; no clinical validation of trigger thresholds.	Trigger ranges may overlap with healthy physiology; response can vary among patients and lesions.	Measure differential release, intact-carrier localization, payload recovery, target engagement, and degradation products.
Biomimetic/edible systems	Immune interaction and local anti-inflammatory activity	Preclinical cell and animal studies [13,14,58,59]; no clinical validation of platform-wide design rules.	Complex composition, batch variability, and cargo attribution complicate translation.	Define composition, potency assay, dose unit, contaminants, stability, and scalable manufacturing controls.

## Data Availability

No new data were created or analyzed in this study. Data sharing is not applicable to this article. All data discussed in the manuscript are derived from previously published sources cited in the article.
